# Introduction to motivational interviewing and its principles.

**DOI:** 10.1192/j.eurpsy.2023.187

**Published:** 2023-07-19

**Authors:** F. Azevedo

**Affiliations:** Psychiatry, Centro Hospitalar de Lisboa Ocidental, Lisbon, Portugal

## Abstract

**Abstract:**

In this second session of our motivational interviewing workshop, we introduce motivational interviewing with its principles. Furthermore, we discuss the pyramid of change and Prochaska’s change model, address the differences between pre-contemplative versus change-resistant patients, and approach different techniques we can use throughout the clinical interview.

**Disclosure of Interest:**

None Declared

